# Influence of Excipients and Spray Drying on the Physical and Chemical Properties of Nutraceutical Capsules Containing Phytochemicals from Black Bean Extract

**DOI:** 10.3390/molecules201219792

**Published:** 2015-12-03

**Authors:** Daniel Guajardo-Flores, Curtis Rempel, Janet A. Gutiérrez-Uribe, Sergio O. Serna-Saldívar

**Affiliations:** 1 Centro de Biotecnología-FEMSA, Tecnológico de Monterrey, Campus Monterrey, Monterrey, Nuevo León 64849, Mexico; jagu@itesm.mx (J.A.G.-U.); sserna@itesm.mx (S.O.S.-S.); 2 Richardson Centre for Functional Foods and Nutraceuticals, Department of Food Science, University of Manitoba, Winnipeg, MB R3T2N2, Canada; cbrempel.sci@gmail.com

**Keywords:** *Phaseolus vulgaris*, spray-drying, maltodextrin, powder flow properties

## Abstract

Black beans (*Phaseolus vulgaris* L.) are a rich source of flavonoids and saponins with proven health benefits. Spray dried black bean extract powders were used in different formulations for the production of nutraceutical capsules with reduced batch-to-batch weight variability. Factorial designs were used to find an adequate maltodextrin-extract ratio for the spray-drying process to produce black bean extract powders. Several flowability properties were used to determine composite flow index of produced powders. Powder containing 6% maltodextrin had the highest yield (78.6%) and the best recovery of flavonoids and saponins (>56% and >73%, respectively). The new complexes formed by the interaction of black bean powder with maltodextrin, microcrystalline cellulose 50 and starch exhibited not only bigger particles, but also a rougher structure than using only maltodextrin and starch as excipients. A drying process prior to capsule production improved powder flowability, increasing capsule weight and reducing variability. The formulation containing 25.0% of maltodextrin, 24.1% of microcrystalline cellulose 50, 50% of starch and 0.9% of magnesium stearate produced capsules with less than 2.5% weight variability. The spray drying technique is a feasible technique to produce good flow extract powders containing valuable phytochemicals and low cost excipients to reduce the end-product variability.

## 1. Introduction

The food, cosmetic and pharmaceutical industries are focusing their interest on different natural compounds as a strategy to enhance the health benefits of their products. Black beans are an example of a good source of bioactive compounds with proven human health benefits [1]. Flavonols and saponins from black beans have been identified as having a proven effect on cancer prevention [2,3,4]. Some of these compounds are highly sensitive to oxidation due to light exposure or temperature. Therefore, characterization of physicochemical properties of plant extracts, processing and formulation are equally important in the product development to stabilize the products, optimize their efficacy and reduce their batch-to-batch variability. Previous studies reported that among 25 ginseng products the product-to-product variability of the concentrations of ginsenosides varied by 15-fold and 36-fold times in capsules and liquids, respectively [5]. The production of functional powders as ingredients is a feasible way to overcome the low bioavailability and degradation of compounds.

Plant extracts can be transformed from liquid to a spray dried powder that includes carriers [6]. The spray drying technique is a powerful tool for delivering cost-effective and high quality ingredients. Spray dried extracts powders are easily transported, handled and reduced in bulk size since they possess good flowability and high stability [7].

Maltodextrin is a water soluble modified starch derivative widely used in food processing as a replacement for synthetic ingredients. They have multifunctional properties such as the bulking and film formation, the ability to bind and to confer protection to bioactive compounds improving the flowability of powders [8] by a significant reduction in the apparent viscosity of feed dispersion. Although this adjuvant has been recommended to produce dry plant extracts via spray-drying, the selection of excipients and concentration for black bean extract powder production cannot be directly inferred from other plant/carrier systems.

Based on the above reasons and considering the lack of scientific literature with regard to black bean extract drying and formulation, the feasibility of the industrial production of black bean extract powders using spray-drying as a processing method was studied. The aim was to obtain spray-dried black bean extract powders with high content of flavonols and saponins. Also, the identification of appropriate excipients to enhance flow characteristics of the spray dried extract were studied in order to deliver uniform doses of the powder during capsule manufacture.

## 2. Results and Discussion

### 2.1. Extract Powder Production

#### 2.1.1. Powder Moisture and Hygroscopicity

The moisture content of the dry black bean extract powders was not significantly different among the treatments (Table 1). The moisture range of 5.2%–5.8% fulfilled the quality parameters for a powder to be considered as a dry product [9] and was within the commonly observed moisture values in industrial spray drying samples (3.9% to 5.9%) [10].

**Table 1:** Moisture content, hygroscopicity and yield of spray dried black bean extract powders obtained from feds with 5% or 10% total solids and different percentages of maltodextrin.

Run	Total Solids in Solution Feed (%)	Maltodextrin in Total Solids (%)	Moisture Content (%)	Hygroscopicity (g/100 g)	Yield (%)
1	5	6	5.8 ± 0.3 a	27.3 ± 2.7 a	78.6 ± 1.4 a
2	5	15	5.7 ± 0.1 a	21.4 ± 1.6 b	77.2 ± 0.6 a
3	5	30	5.5 ± 0.2 a	19.6 ± 1.1 b	20.6 ± 3.8 d
4	5	45	5.3 ± 0.1 a	22.1 ± 1.3 b	18.0 ± 5.7 d
5	10	60	5.6 ± 0.0 a	17.6 ± 1.8 c	77.4 ± 0.5 a
6	10	80	5.2 ± 0.1 a	17.0 ± 1.2 c	46.9 ± 4.3 c
7	10	90	5.4 ± 0.1 a	13.5 ± 1.4 d	66.5 ± 2.9 b

Different letters by column indicate significant differences estimated by Tukey Tests (*p* < 0.05).

Powders obtained by spray drying at 5% total solids solution had higher hygroscopicity than those recovered from a 10% total solids solution (Table 1). Additionally, the percentage of maltodextrin used in the spray drying process affected the ability of the materials to attract and absorb the moisture from the surrounding environment. Dry black bean extract powder obtained by addition of 90% maltodextrin showed the lowest hygroscopicity of 13.5 ± 1.4 g/100 g. As the maltodextrin concentration decreased, an increase in hygroscopicity was observed as previously reported in other plant extracts powders [7,11,12,13].

The lowest yield was obtained from runs 3 and 4 with 20.6% and 18.0%, respectively. The highest yields were from runs 1, 2 and 5 with 78.6%, 77.2% and 77.4%, respectively (Table 1). These values were more than acceptable for lab-scale spray dryers [13] and corresponded to runs made with 5% of total solids combined with 6 or 15% of maltodextrin or 60% maltodextrin in a fed with 10% total solids. When the maltodextrin: black bean extract ratio was exceeded, a thin layer formed on the chamber walls and thus decreased the process efficiency as in runs 3, 4 and 6.

#### 2.1.2. Powder Phytochemical Characterization

Myricetin-3-*O*-glucoside, soyasaponin Ba and soyasaponin αg (2.1, 2.6 and 7.4 mg/100 g DB, respectively) were the main compounds identified in the lyophilized black bean extract and spray dried black bean extract powders (Table 2). A complete recovery of myricetin-3-*O*-glucoside, soyasaponins Ba and αg in the dry powders was obtained in the case of extract prepared by adding 6% maltodextrin (Run 1). Despite the fact Run 2 had a similar yield as Run 1, the compound recovery was lower, especially for flavonols. Further addition of maltodextrin reduced the recovery of phytochemicals from black bean extract powders, even for Run 5 that was also among those with the highest yield. These results showed that adding 6% maltodextrin in the spray drying feed with 5% of total solids led to an efficient sealing of the dry powders surface and therefore efficient preservation of black bean phytochemicals.

**Table 2:** Phytochemical composition of lyophilized black bean extract and recovery percentage after spray drying using different amounts of total solids and maltodextrin.

Phytochemical	λ_max_ Absorption		[M + H]^+^ (*m*/*z*)	Content (mg/100 g DB)	Recovery of Phytochemicals after Spray Drying (%): Run						
					1	2	3	4	5	6	7
Myricetin-3*O*-glucoside	260	358	481	2.1 ± 0.1	100	61	100	67	84	79	69
Quercetin-3*O*-glucoside	258	356	465	0.8 ± 0.2	100	69	54	43	51	12	27
Kaempferol-3*O*-glucoside	266	348	449	0.6 ± 0.1	100	74	63	47	56	78	47
Myricetin	256	274	319	1.3 ± 0.2	56	33	42	59	31	77	88
Quercetin	255	371	303	1.4 ± 0.2	75	79	48	ND	ND	83	41
Soyasaponin Ba	-	-	959	2.6 ± 0.4	73	87	ND	ND	ND	52	ND
Kaempferol	266	366	287	0.7 ± 0.1	100	93	48	27	58	74	ND
Soyasaponin αg	295	-	1085	7.4 ± 0.8	100	59	38	23	38	69	ND

ND: not detected.

### 2.2. Evaluation of the Physical Properties and Flowability of Excipients, Black Bean Extract Powders and Formulations for Placebo Capsule Production

Starch exhibited the highest particle size followed by microcrystalline cellulose 50 > black bean spray dried powder > maltodextrin > microcrystalline cellulose 25 (Table 3). Particles smaller than 100 μm are considered to be cohesive, however they could increase the powder flowability if they adhere to the surfaces of larger particles thus smoothing out the surface structure [14].

**Table 3:** Physical properties of different materials used.

Materials	Particle Size (μm) ^1^	Bulk Density (g/cm^3^)	Tapped Density (g/cm^3^)	True Density (g/cm^3^) ^2^
Black bean spray dried powder	49	0.6 ± 0.0	0.83 ± 0.3 a	n.d.
Maltodextrin	30–40	0.5 ± 0.1	0.63 ± 0.2 b	1.50
Microcrystalline Cellulose 25	25	0.6 ± 0.1	0.89 ± 0.2 a	1.56
Microcrystalline Cellulose 50	50	0.6 ± 0.1	0.84 ± 0.2 a	1.43
Starch	60-90	0.5 ± 0.1	0.58 ± 0.1 c	1.48

^1^ Parameter values defined by supplier, with the exception of black bean spray dried powder, which particle size was defined experimentally according to the d (0.5); ^2^ Parameter values defined by supplier, the black bean spray dried powder was not determined due to the lack of a pycnometer equipment. Different letters by column indicate significant differences estimated by Tukey Tests (*p* < 0.05).

Among the powders tested without starch, formulations containing microcrystalline cellulose 50 and the low level of magnesium stearate exhibited fair flow powder properties (Table S1, Supplementary Material). The addition of starch into the powders as a replacement of maltodextrin and microcrystalline cellulose improved the composite flow index (CFI) (Table S2, Supplementary Material).

The powder containing 50% maltodextrin, 49.1% of microcrystalline cellulose 50 and 0.9% of magnesium stearate but no starch (Powder A) resulted in a CFI of 52.8% (Table 4). In contrast, the powder containing 25.0% of maltodextrin, 24.1% of microcrystalline cellulose 50, 50.0% of starch and 0.9% of magnesium stearate (Powder B) and the powder containing 25.0% maltodextrin, 74.1% starch and 0.9% magnesium stearate (Powder C), exhibited CFIs of 60.5% and 65.0%, respectively, and a capsule weight variability of less than 2.5%. The maltodextrin and starch low tapped densities (Table 3) allowed the particles to rearrange and increase the flowability [15].

**Table 4:** Effect of the powder composition on its physical properties, flowability and capsule weight.

		Treatment		
		A	B	C
Composition (%)	Maltodextrin	50	25	25
	MCC 50	49.1	24.1	-
	Starch	-	50	74.1
Particle size distribution (μm)	d(0.1)	22.51 b	26.39 a	22.95 a
	d(0.5)	57.55 a	45.51 b	43.71 b
	d(0.9)	135.6 a	76.81 b	81.27 b
Surface area	(m^2^/g)	0.07	0.06	0.06
Powder flowability	CFI (%)	52.8 c	60.5 b	65.0 a
	Flow category	Fair	Fair	Good
Capsule	Weight (mg)	565.9 c	728.6 a	614.1 b
	Weight variability (%)	0.5	1.5	1.5

MCC 50, Microcrystalline cellulose 50; CFI, Composite flow index. Different letters by column indicate significant differences estimated by Tukey Tests (*p* < 0.05).

### 2.3. Black Bean Powder Capsule Production

**Figure 1:**
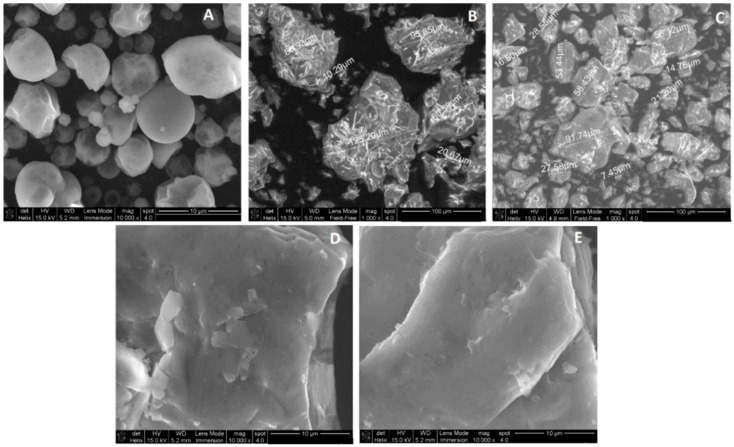
(**A**) Scanning electron microscopy (SEM) analysis at 10,000× of lyophilized black bean extract, and (**B**,**C**) black bean capsule formulations using 25% of maltodextrin with 24.1% of microcrystalline cellulose 50, 50% of starch and 0.9% of magnesium stearate (powder B) or with 74.1% of starch and 0.9% of magnesium stearate as excipients (powder C) and (**D**,**E**) at 1000× for powder B and C.

Formulations A, B and C were adjusted to achieve 200 mg of black bean extract per capsule which corresponded to 73.75%, 57.25% and 68.35% of total weight, respectively. The CFI of all formulations was significantly reduced due to the hygroscopicity of black bean spray dried powder (Table 5). Lyophilized black bean extract had a particle size lower than 10 um (Figure 1A) but it interacted with the maltodextrin, microcrystalline cellulose 50 and starch to form new complexes of larger particle size (Figure 1B,C). Particle size distribution d(0.9) of powder obtained from formulation C was the only one that diminished after the addition of the black bean extract powder (Table 5). In comparison with powder B, a higher content of starch instead of microcrystalline cellulose 50 in powder C allowed the inter-particulate interactions that have been correlated to flow indicators [16]. Also, the interaction between starch and black bean extract powder formed not only bigger particles but with a rougher structure. These structures reduced the powder agglomeration and thus improved the CFI scores (Figure 1B,C).

The best CFI score was obtained from powder B (55.1%), whose flow can be considered as fair. The powders A and C had passable flow properties with a CFI of 47.8% and 48.3%, respectively and contained more densely packed particles than powder B, thus significantly reducing the powder ability to flow freely [17]. Thus, and interaction between starch and microcrystalline cellulose 50 improves the powder flowability compared to starch and microcrystalline cellulose 50 excipients by themselves even at high level of composition (Tables S1 and S2, Supplementary Material).

The capsule weight ranged from 348.3 to 456.6 mg, and contained from 192.3 to 200.7 mg of black bean extract in each capsule (Table 5). The weight variability in all formulations tested exceeded the 2.5% required, particularly the powder C had the highest capsule weight variability (7.4%). The drying process prior to the capsule production significantly reduced the weight variability (Table 5). In particular, the larger size of particles found in powder B than in powder C (Figure 1D,E) affected the cohesiveness and friction forces of the powder. Previous studies demonstrated that bigger granules containing starch, revealed better flow cohesivity than smaller granule size with high starch content [18]. The microcrystalline cellulose 50 may act as a glidant to facilitate the flow of granules. Despite powder B, did not contain 200 mg of extract, it was the only with a capsule weight variability of less than 2.5%. The amount of extract can be easily modified by increasing the black bean spray dried powder in the formulation.

**Table 5:** Physical properties and flowability of black bean formulation and the effect of a drying prior encapsulation on capsule weight and black bean extract content.

		Treatment		
		A ^1^	B ^2^	C ^3^
Particle size distribution (μm)	d(0.1)	13.58	16.87	4.06
	d(0.5)	57.29	57.08	28.05
	d(0.9)	138.27	139.3	61.84
Surface area	(m^2^/g)	0.09	0.09	0.21
Powder flowability	CFI (%)	47.8	55.1	48.3
	Flow category	Passable	Fair	Passable
Capsules using powder without previous drying	Weight (mg)	348.3 c	456.6 a	391.7 b
	Weight variability (%)	3.1	3.0	7.4
	Black bean extract amount (mg)	192.3 b	196.8 ab	200.7 a
	Black bean extract variability (%)	6.0	5.9	14.8
Capsules using powder with previous drying	Weight (mg)	395.3 c	457.2 a	417.6 b
	Weight variability (%)	2.7	1.6	2.5
	Black bean extract amount (mg)	218.4 a	196.2 b	214.0 a
	Black bean extract variability (%)	5.5	3.2	5.8

^1^: Black bean capsule formulation using 50.0% of maltodextrin, 49.1% of microcrystalline cellulose 50 and 0.9% of magnesium stearate as excipients; ^2^: Black bean capsule formulation using 25.0% of maltodextrin, 24.1% of microcrystalline cellulose 50, 50% of starch and 0.9% of magnesium stearate as excipients; ^3^: Black bean capsule formulation using 25.0% of maltodextrin, 74.1% of starch and 0.9% of magnesium stearate as excipients.

## 3. Experimental Section

### 3.1. Materials

Black bean extract was obtained from the common bean cultivar “Negro San Luis” supplied by a local dealer. The germination process was carried out as described in previous reports [19]. After one-day germination, seeds were dehydrated in an oven set at 60 °C for 4 h and then dissected into sprouts, seed coats and cotyledons. Only sprouts and seed coats were weighed, ground into a powder (GX4100 Coffee and Spice Grinder, Krups, Monterrey, Mexico).

### 3.2. Extraction Process

Black bean extract was produced at the Centro de Biotecnología-FEMSA at Tecnológico de Monterrey Campus Monterrey, N. L. Mexico. Phytochemical extraction was performed according to previous reports [19]. Sprouts and seed coats (600 g) were combined with 6 L of 80% aqueous methanol. The process was carried out in a 20 L reactor (Chemglass, Vineland, NJ, USA) for 3 h at 200 rpm and 25 °C. Black bean sprouts and seed coats were washed five times. For analysis purposes of determining the phytochemical composition and further recovering after the subsequent process, the liquid extract was lyophilized (Virtis freezemobile Sentry 2.0, Gardiner, NY, USA) after filtration using a No. 1 Whatman filter paper and methanol evaporation under vacuum at 60 °C in a rotavapor (Buchi R.210, New Castle, DE, USA). The powder was stored at −80 °C until further analysis to avoid the phytochemical degradation.

### 3.3. Analysis of Powder Moisture Content and Hygroscopicity

Moisture content and hygroscopicity were analyzed using the standard procedure described in previous reports [7]. The sample was dried at 105 °C for 3 h. Samples of each powder (1 g) were placed at 25 °C in a desiccator filled with NaCl saturated solution (70% RH). The hygroscopicity was measured after 10 days and expressed as the absorbed water (weight in g) per 100 g of dry extract powder.

### 3.4. Spray drying Using Different Solutions of Black Bean Extract with Maltodextrin

The feed solids concentration was adjusted to 5% or 10% (*w*/*v*). Maltodextrin (DE100) was used as a carrier material in the liquid feed at different dry mass percentages of total feed solids (5%, 15%, 30%, 45%, 60% or 80%). Maltodextrin was dissolved in distilled water and then mixed with the different percentages of black bean extract using a magnetic stirrer at 25 °C for 15 min. The prepared liquid feed was spray dried (Pruvis Mini Spray GB201-A; Yamato, Santa Clara, CA, USA). The process inlet temperature ranged from 170 to 180 °C and the outlet air temperature ranged from 60 to 65 °C. The atomization volumetric flow rate was 0.5 m^3^/min. The feed volumetric flow rate was 4.2 mL/min. The drying air pump was fixed at 0.13 MPa. The yield of the spray drying process was determined gravimetrically according to the mass of total solids measured in the feed and the mass of the dry powder obtained at the end of the process. Yield was expressed as a percentage of the mass of final product compared to the total amount of the spray dried materials.

### 3.5. Determination of Flavonols and Saponins

The contents of flavonoids and saponins in the lyophilized and dry black bean extract powders were determined using a HPLC-DAD-ELSD (Agilent Technologies, Santa Clara, CA, USA) system according to previous reports [19]. The extracts were resuspended in 80% methanol at 1 mg/mL prior their injection to the HPLC. Elution was conducted with (A) HPLC-grade water adjusted to pH 2 with trifluoroacetic acid (Sigma, St. Louis, MO, USA) and (B) HPLC-grade acetonitrile. Separation was achieved with 20% B for the first 6 min, increasing the B concentration to 50% at 12 min and to 100% at 30 min. Flavonoids were quantified using authentic standards of aglycones. All saponins were quantified using the evaporative light scattering detector (ELSD) and a standard curve obtained from soyasaponin I purified in our laboratory [19]. To confirm the presence of the 2,3-dihydro-2,5-dihydroxy-6-methyl-4*H*-pyran-4one (DDMP) conjugated saponins, their detection using UV absorption maximum at 295 nm was obtained. The content of each phytochemical in the dry extract powders was expressed as percentage of recovery compared to lyophilized powder extracted range contents.

### 3.6. Excipient Formulation and Capsule Manufacture

All the capsule formulation components had pharmaceutical and national formulary (NF) grade. Gelatin capsules were obtained from Capsugel, Inc. (Bornem, Belgium), and black bean capsules were prepared and formulated under GMP conditions at the Richardson Centre for Functional Foods and Nutraceuticals at the University of Manitoba, Winnipeg, MB, Canada. Maltodextrin were obtained from Cargill (Minneapolis, MN, USA), microcrystalline cellulose and starch from DFE-pharma (Princeton, NJ, USA) and magnesium stearate from Min-Chem Inc. (Oakville, ON, Canada).

Different formulation powders (50 g) were prepared containing different levels of maltodextrin, magnesium stearate as lubricant and microcrystalline cellulose (Table S1, Supplementary Material). Only one type of microcrystalline cellulose (25 or 50) was used per formulation. A second experimental design was used to assess the effect of starch at different levels of content on the powder properties as an economically substitution to microcrystalline cellulose and maltodextrin, magnesium stearate remained fixed at 0.9% (Table S2, Supplementary Material).

Formulations were blended for 2 to 5 min using a hand blender (SM9617, Severin, Sunderm, Germany) and powder flowability traits were evaluated immediately after the mixing process. Evaluations were carried out in a room with controlled temperature (22 °C ± 5 °C) and relative humidity (38% ± 5%) in order to avoid environmental effects over excipient flow properties. Additional drying process was applied to powder to improve the weight variability of capsules using a Memmert oven (Eagle, WI, USA) at 40 °C for 6 h. All the powders were encapsulated in Size #0 natural transparent hard capsule.

### 3.7. Bulk and Tapped Density Determination

The percentage of compressibility, also known as Carr´s Index, was calculated from the bulk and tapped density of a powder. The bulk (ρbulk) and tapped (ρtap) densities were determined according to the method described in the US Pharmacopeia USP XXIV [20]. A 100-mL glass cylinder was filled gently with 50 g of powder to measure the volume and the bulk density ρbulk was calculated dividing the mass by the volume. The same measurement was repeated after 360 taps to measure tap density ρtap. Carr´s compressibility index (CI) provides an indirect measurement of flow, and was obtained using equation:

CI = (100 × (ρbulk − ρtap))/ρbulk
(1)

### 3.8. Powder Flowability Analysis

Critical orifice diameter (COD) studies were performed using a Flowdex Powder Flowability Tester (Hanson Research Corporation, Northridge, CA, USA). For each sample, 50 g of powder was introduced into a flat-based cylindrical hopper fitted with a series of plates having orifices in the diameter range 4–34 mm. After loading, the powder was allowed to stand for 1 min before opening the orifice surface shutter at the base of the hopper. The COD was defined as the diameter of the smallest orifice through which the powder flowed.

The angle of repose (AR) was determined by measuring the cone height *vs.* the base formed by pouring 50 g of powder falling through a stainless steel funnel placed from a height of 5 cm from the table surface until a stable cone was produced. The angle of repose was established as described by Taylor, Ginsburgh, Hickey and Gheyas [14] using the equation:

tan θ = Height/(0.5 × Base)
(2)

A composite flow index (CFI) for the pharmaceutical powders was calculated according to the United States Pharmacopeia Convention [20] to include all different flow tests.

(3) CFI=COD × 0.5+CI × 0.4+AR × 0.1

### 3.9. Capsule Weight Uniformity

Hard gelatin capsules formulations were prepared using a CAP8 Capsule Filling Machine (Capsugel, Greenwood, SC, USA). Right after the blending process, 50 g of powder was poured into the filler machine to produce small batches of 180 capsules. Capsule weight uniformity was measured by weighting each capsule on the analytical balance. Placebo capsules were first produced in order to analyze the excipients effect on weight variability. Then, the intended goal was to produce a black bean capsule with approximately 200 mg of black bean extract powder using the best placebo treatments, to obtain a weight variability lower than 2.5%. The capsules weight variability was compared to the requirement of acceptance according to the USP Convention [20].

### 3.10. Particle Morphology, Particle Size and Surface Area Distributions

The methanolic black bean extract and the best black bean powders were lyophilized for particle shape analysis using a scanning electron microscopy (SEM) NanoSEM-200 (FEI Company, Hillsboro, OR, USA). Particle size analysis and surface area distributions were conducted with a Malvern Mastersizer 2000 (Malvern Instruments Ltd, Malvern, UK) fitted with a hydro 2000SM wet accessory. A volume of 10 mL of distilled water was used as dispersant for all the materials. Prior to every quantitation, background measurements were taken. After completion of the background measurement, the powders were added to render an obscuration of 20% ± 5% to obtain the particle size measurement.

### 3.11. Statistical Analysis

Statistical analyses were conducted using the JMP^®^ Version 5 software (SAS Institute Inc., Cary, NC, USA) and differences among means were compared with Tukey’s tests with a level of significance of *p* < 0.05. All analyses were done in triplicate and results were expressed as mean ± standard deviations.

## 4. Conclusions

It can be concluded that the retention of flavonoids and saponins from black bean extract powder decreases with the increase of maltodextrin concentration in the spray drying feed. The physical properties of starch and maltodextrin such as low bulk and tapped densities and relatively high particle size affected positively the flow properties of obtained powders. The composite flow index of all powder formulations was significantly reduced due to the hygroscopicity of black bean spray dried powder. The new complexes formed by the interaction of black bean powder with maltodextrin, microcrystalline cellulose 50 and starch exhibited not only bigger particles but with a rougher structure than using only maltodextrin and starch as excipients. The drying process prior to the production of capsules significantly improved the flow of the powders within the capsule filling machine, resulting in an increase in the total weight of the capsules and a reduction in weight variability. The black bean capsule formulation containing a blend of 25.0% of maltodextrin, 24.1% of microcrystalline cellulose 50, 50% of starch and 0.9% of magnesium stearate exhibited the best flowability properties to produce capsules with less than 2.5% batch-to-batch variability. This study demonstrated the feasibility of producing black bean capsules using the spray drier technique to produce powders without the degradation of the phytochemicals and with better flowability properties, thus reducing the end-product variability.

## Supplementary Materials

Additional material can be obtained free of charge via E-Mail: danielgdo@itesm.mx. Supplementary materials can be accessed at: http://www.mdpi.com/1420-3049/20/12/19792/s1.
