# Supplementary material for: Influence of Excipients and Spray Drying on the Physical and Chemical Properties of Nutraceutical Capsules Containing Phytochemicals from Black Bean Extract
Source: Molecules. 2015 Dec 3;20(12):21626–35. doi: 10.3390/molecules201219792 (PMC6332071; doi:10.3390/molecules201219792)
Supplement: Supplementary file 1 [file molecules-20-19792-s001.pdf]

Supplementary Materials: Influence of Excipients and Spray Drying on the Physical and Chemical Properties of Nutraceutical Capsules Containing Phytochemicals from Black Bean Extract

Daniel Guajardo-Flores, Curtis Rempel, Janet A. Gutiérrez-Uribe and Sergio O. Serna-Saldívar

1. Flow Properties of Powders

1.1. Angle of Repose

According to USP 30-NF 25 [1], powder flow with repose angles between 25–30° powder flow is excellent, among 31–35° the flow is good and within 36–40° the flow is fair. For values higher than 41°, the powder has bad flow properties. The best angle of repose was shown by powder obtained from tests 4 and 6 (33 ± 2° and 33 ± 1° in Table S1), whose flow can be considered good. Moreover, the powders obtained from experiments 3 and 7 presented angles of repose of 40°± 1° and 38°± 0°, and, thus, fair flowability. The effect of solids concentration on the angle of repose is in agreement with the results reported by others studies using maltodextrin mixtures as carriers [2]. In previous studies using the angle of repose test, powders with strong structural strength will form a big pile when dispersed in a container resulting in a low bulk density; whereas a structurally weak powder will collapse easily exhibiting a high bulk density [3].

The addition of starch into the powder formulations significantly reduced the angle of repose (Table S2). The test S.6 containing 25.0% maltodextrins, 74.1% starch and 0.9% magnesium stearate exhibited an improved angle of repose of 28 ± 1°, whose flow can be considered good. The improvement in the angle of repose could be attributed to the particle size and shape of the starch and a reduction in the frictional force between the individual particles. The higher particle size of starch could reduce the ability to interact with the glidant and the small particles of the materials affecting the cohesiveness and friction forces [4].

Table S1. Experimental matrix design and studied flowability parameters of formulations.

Run	MD (g)	MCC 25 (g)	MCC 50 (g)	MS (g)	Angle of Repose (°)	CI (%)	COD (mm)	CFI (%)	Flow Category
1	25	24.75	0	0.25	56 [a]	35.9 [a]	22	26.0 [e]	Poor
2	25	0	24.55	0.45	48 [c]	30.4 [b]	24	30.7 [e]	Poor
3	25	0	24.75	0.25	40 [d]	24.9 [c]	14	52.8 [a]	Fair
4	25	24.55	0	0.45	33 [e]	25.4 [c]	18	47.8 [b]	Passable
5	37.5	12.25	0	0.25	53 [b]	24.2 [c]	20	41.7 [c]	Passable
6	37.5	0	12.05	0.45	33 [e]	25.2 [c]	20	45.2 [b]	Passable
7	37.5	0	12.25	0.25	38 [d]	25.6 [c]	14	52.7 [a]	Fair
8	37.5	12.05	0	0.45	43 [d]	26.0 [c]	24	36.1 [d]	Passable

MD.- Maltodextrin; MCC 25, microcrystalline cellulose 25; MCC 50, microcrystalline cellulose 50, MS, magnesium stearate; CI, composite index; COD, Critical orifice diameter; CFI, Composite flow index. Different letters indicate significant differences by column, by the Tukey test ($p < 0.05$). Different flow category according to Horn (2008).

Table S2. Experimental matrix design using starch as an alternative excipient.

Run	MD (g)	MCC 25 (g)	MCC 50 (g)	S (g)	Angle of Repose (°)	CI (%)	COD (mm)	CFI (%)	Flow Category	CWV (%)
S.1	25	0	12.125	12.425	35 1 [b]	27.5 [a]	16	48.3 [d]	Passable	4.0
S.2	25	12.125	0	12.425	44 [a]	27.2 [a]	24	34.7 [e]	Poor	-
S.3	25	0	0	24.55	31 [c]	21.0 [b]	12	61.7 [b]	Fair	4.9
S.4	12.5	0	12.125	24.925	31 [c]	22.5 [b]	12	60.5 [b]	Fair	1.5
S.5	12.5	12.125	0	24.925	36 [d]	22.0 [b]	14	56.7 [c]	Fair	5.0
S.6	12.5	0	0	37.05	28 [c]	21.4 [b]	10	65.0 [a]	Good	0.5

MD.- Maltodextrin; MCC 25, microcrystalline cellulose 25; MCC 50, microcrystalline cellulose 50, MS, magnesium stearate; CI, composite index; COD, Critical orifice diameter; CFI, Composite flow index; CWV, capsule weight variability determined by the weight measures of 180 capsules. Different letters indicate significant differences by column, by the Tukey test ($p < 0.05$). Different flow category according to Horn [5].

1.2. *Carr's Compressibility Index*

Carr's compressibility indexes (CI) of 10% indicate excellent flow, between 11% and 15% they indicate good flowability, between 16% and 20% the powder flow is fair, between 21% and 25% the product has acceptable flow properties and between 26% and 31% the powder flow is poor [2]. According to Table S2, the highest CI was shown in samples 1 and 2 which had a poor flow (27.5% ± 1.2% and 27.2% ± 1.1%). Experiments 3 and 7 showed fair flow with CI values between 24.9% ± 3.0% and 25.6% ± 2.1%, respectively. By the addition of starch as an excipient for the powder formulation, the compressibility index ranged from 21.0% ± 1.8% to 27.5% ± 1.2% (Table S2). The interaction between high levels of starch with maltodextrins and reduced amount of microcrystalline cellulose resulted in an acceptable flow powder properties. The extent of volume reduction in a powder bed during transport and handling of powder, simulated by tapping, is an indication of the cohesiveness of the powder and frictional forces between individual particles in the bed. The particles are forced to rearrange and lose contact with each other for a moment during tapping and flowing through the funnel, thereby improving the packing which coincides with a reduction in the volume of the powder bed [6].

1.3. *Critical Orifice Diameter*

The critical orifice diameter (COD) was defined as the diameter of the smallest orifice through which the powder flowed. There is no index available for COD to distinguish between excellent, good, average and poor flowing powders. However, the critical orifice diameter has recently been correlated to the particle size, densities and surface properties of a mixed powder [7]. As shown in Table S1, the best COD was shown by the powder obtained from experiments 3 and 7 which contains MCC 50 instead of MCC 25 (COD = 14). As expected, the incorporation of starch into the formulation of powders, improved the flowability powders by reducing the COD (Table S2). The best COD was shown by powder obtained from experiment S.6 (COD = 10) followed by the powders obtained from experiments S.3, S.4 and S.5 (COD = 12, 12 and 14, respectively).

1.4. *Composite Flow Index*

The composite flow index (CFI) is a parameter that integrates the powder flowability properties in just a single parameter. Tables S1 and S2 show the CFI of each tested powder based on their flowability properties and describes its flowability behavior. Among the powders tested without starch, the experiments 3 and 7 had a CFI of 52.8% and 52.7%, whose flow can be considered fair. The addition of starch into the experiments formulation improved the CFI (Tables S1 and S2). The best CFI was obtained from experiment S.6 which with a CFI of 65.0%, which is classified as having good flow properties. The powders obtained from experiments S.3, S4 and S.5 presented a fair flow CFI scoring of 61.7%, 60.5% and 56.7%, respectively.

1.5. Capsule Weight Variability

The capsule weight variability ranged from 0.5% to 5.0%. The lowest variation in capsule weight were obtained from experiments 3, S.4 and S.6 (1.5%, 1.5% and 0.5%, respectively). No major correlations between material attribute CFI and weight variability of the field capsules were observed ($p > 0.05$), suggesting that weight variability is affected depending the type and amount of excipients used in the formulations. Also, the process parameters such as the filling speed, compression ratio and volume of the dossier chamber could have an impact on the weight variability of the filled capsules [8].

References

1. United Sates Pharmacopeial Convention (USP). *The United States Pharmacopeial Convention USP 32-NF 27*; USP: Rockville, MD, USA, 2007 p. 4134.
2. Moreira, G.E.G.; Maia Costa, M.G.; Rodriguez de Souza, A.C.; Sousa de Brito, E.; Dantas de Medeiros, M.F.; Azeredo, H.M.C. Physical properties of spray dried acerola pomace extract as affected by temperature and drying AIDS. *LWT Food Sci. Technol.* **2009**, *42*, 641–645.
3. Harnby, N.; Hawkins, A.E.; Vandame, D. The use of bulk density determination as a means of typifying the flow characteristics of loosely compacted powders under the conditions of relative humidity. *Chem. Eng. Sci.* **1987**, *42*, 879–888.
4. Chang, C.K.; Alvarez-Nunez, F.A.; Rinella, J.V., Jr.; Magnusson, L.; Sueda, K. Roller compactation, granulation and capsule product dissolution of drug formulations containing a lactose or mannitol filler, starch and talc. *AAPS PharmSciTech* **2008**, *9*, 597–604.
5. Horn, E. Deveolpment of a Composite Index for Pharmaceutical Powders. Master's Thesis, North-West University, Mahikeng 2790, South Africa, 2008.
6. Adbullah, E.C.; Geldart, D. The use of bulk density measurements as flowability indicators. *Powder Technol.* **1999**, *102*, 151–165.
7. Miccio, F.; Barletta, D.; Poletto, M. Flow properties and arching behavior of biomass particulate solids. *Powder Technol.* **2013**, *235*, 312–313.
8. Faulhammer, E.; Llusa, M.; Radeke, C.; Scheibelhofer, O.; Lawrence, S.; Biserni, S.; Calzolari, V.; Khinast, J.G. The effects of material attributes on capsule fill weight and weight variability in dosator nozzle machines. *Int. J. Pharm.* **2014**, *471*, 332–338.
